# The One Health Approach is Necessary for the Control of Rift Valley Fever Infections in Egypt: A Comprehensive Review

**DOI:** 10.3390/v11020139

**Published:** 2019-02-06

**Authors:** Mohamed Fawzy, Yosra A. Helmy

**Affiliations:** 1 Department of Virology, Faculty of Veterinary Medicine, Suez Canal University, Ismailia 41522, Egypt; mohamed_mohamed4@vet.suez.edu.eg; 2 Food Animal Health Research Program, Department of Veterinary Preventive Medicine, Ohio Agricultural Research and Development Center, The Ohio State University; Wooster, 44691 OH, USA; 3 Department of Animal Hygiene, Zoonoses and Animal Ethology, Faculty of Veterinary Medicine, Suez Canal University, Ismailia 41522, Egypt

**Keywords:** Rift Valley Fever (RVF), One Health approach, zoonotic virus, vaccine, Egypt

## Abstract

Rift Valley fever (RVF) is an emerging transboundary, mosquito-borne, zoonotic viral disease caused high morbidity and mortality in both human and ruminant populations. It is considered an important threat to both agriculture and public health in African and the Middle Eastern countries including Egypt. Five major RVF epidemics have been reported in Egypt (1977, 1993, 1994, 1997, and 2003). The virus is transmitted in Egypt by different mosquito’s genera such as *Aedes*, *Culex*, *Anopheles*, and *Mansonia*, leading to abortions in susceptible animal hosts especially sheep, goat, cattle, and buffaloes. Recurrent RVF outbreaks in Egypt have been attributed in part to the lack of routine surveillance for the virus. These periodic epizootics have resulted in severe economic losses. We posit that there is a critical need for new approaches to RVF control that will prevent or at least reduce future morbidity and economic stress. One Health is an integrated approach for the understanding and management of animal, human, and environmental determinants of complex problems such as RVF. Employing the One Health approach, one might engage local communities in surveillance and control of RVF efforts, rather than continuing their current status as passive victims of the periodic RVF incursions. This review focuses upon endemic and epidemic status of RVF in Egypt, the virus vectors and their ecology, transmission dynamics, risk factors, and the ecology of the RVF at the animal/human interface, prevention, and control measures, and the use of environmental and climate data in surveillance systems to predict disease outbreaks.

## 1. Introduction

Rift Valley Fever (RVF) is an acute, arthropod-borne viral disease of sheep, goats, cattle, camels, and humans caused by single-stranded ambisense RNA virus, that belongs to the genus *Phlebovirus*, the family *Phenuiviridae* and the order *Bunyavirales* [1,2,3]. The virus genome is tripartite RNA that contains three segments; large (L), medium (M) and small (S) [4]. The L-segment encodes the viral polymerase (L protein) [5]. The S-segment translates into nucleoprotein and a non-structural protein (*NSs*) that determine the virus virulence [6,7]. The M-segment encodes two envelope glycoproteins, Gn and Gc, and two uncharacterized polypeptides (NSm1, and NSm2) [8,9].

Rift Valley Fever disease was first identified in sheep in the Rift Valley province of Kenya in 1931 from which it was named [10]. RVF is considered to be one of the most important zoonotic viral diseases in Egypt. The disease is endemic in sub-Saharan African countries. It has caused epizootics in multiple countries including Egypt, Saudi Arabia, Yemen, South Sudan, Kenya, Tanzania, Madagascar, Somalia, Mauritania, and Senegal [11]. RVF has a great economic impact on meat and dairy products, and also caused abortion storms in pregnant animals. In 2007 incursions of RVF in Kenya were estimated to have cost $32 million in US Dollars, due to livestock death, closure of livestock markets and reduced sales of animal products [12]. In 2000, the estimated cost during the first RVF outbreak in the Arabian Peninsula was estimated as $90 million US Dollars [13,14].

RVF virus (RVFV) is transmitted to humans through drinking unpasteurized milk, direct contact with infected animal’s blood, aerosol or the bite of infected mosquitoes [15]. *Aedes* and *Culex* mosquitoes are considered the main vectors. *Aedes* mosquitoes are also considered as reservoirs [11]. Fortunately, RVFV is not often transmitted from human-to-human, however, aerosol transmission of RVFV is suspected to have occurred among close contacts, which may have contributed to nosocomial infections and community outbreaks [16]. Disease in humans varies from influenza-like illness to more complicated forms such as hemorrhagic fever, retinitis, renal failure, encephalitis, and miscarriage [17,18]. Notably, RVF outbreaks are episodic and associated with climatic, hydrologic and socioeconomic factors [19]. During RVF outbreaks, significant numbers of infected human cases have been documented, leading to healthcare challenges. Several RVF outbreaks have been recorded in Egypt with devastating morbidity in humans such as the 1977 outbreak (200,000 human cases and 600 deaths) [15]. Human epidemics are often preceded by epizootics in livestock. Hence, controlling RVF in animals is thought to be effective in preventing human disease through disrupting the transmission cycle [20]. Currently, the One Health approach, an integrated approach for the understanding and management of animal, human, and environmental determinants of disease, is used in part for the prevention and control of RVF infection and transmission [18,21]. This approach is thought to be very strategic in that risk factors for RVF transmission are increasingly recognized to be interlinked: expansion of human and animal populations, ecological changes, climate variations, etc. Hence, there is a critical need for close One Health-oriented collaborations among professionals working in diverse sectors such as animal health, human health, public health, entomology, and animal production. In this review, we have sought to review the history of the RVF disease, the previous outbreaks in Egypt, transmission dynamics, disease in humans and animals, epidemiological risk factors related to the introduction and spread of RVF in Egypt, surveillance for RVF, employment of vaccines against RVFV infection, and the role of One Health approach in the prevention and control of RVF. We posit that One Health efforts to control RVF in Egypt can be expanded with strong potential for added preventive effect.

## 2. Taxonomy, Morphology and Genome Organization of RVF Virus

**Figure 1:**
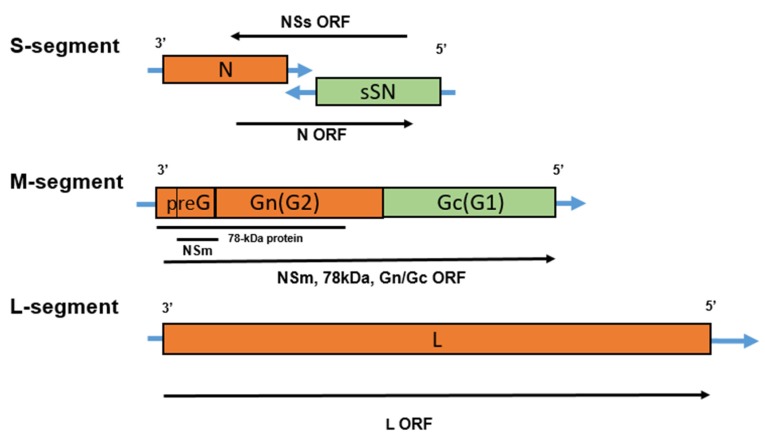
The RVF virus tripartite RNA genome structure. S-segment encodes for nucleoprotein (N) and a non-structural protein (*NSs*) that determine the virus virulence. M-segment encodes two envelope glycoproteins, Gn and Gc, and two uncharacterized polypeptides (NSm1, and NSm2). L-segment encodes L protein (viral polymerase).

RVFV is single-stranded segmented RNA belongs to the genus Phlebovirus, family Phenuiviridae of order Bunyavirales. The family Phenuiviridae includes 4 genera: Phlebovirus, Goukovirus, Phasivirus, and Tenuivirus [22]. The RVF virus is enveloped with ribonucleocapside core, is spherical or pleomorphic in shape, 80–120 nm in diameter with surface glycoprotein projections of 5–10 nm, and embedded in a lipid bilayer envelope [9]. The RVF virus contains a thick linear viral ribonucleocapsid of 2–2.5 nm in diameter, 200–300 nm in length and displays helical symmetry [9]. The RVF virus genome contains a single strand tripartite with L, M and S-segments [3,23] as previously shown in Ikegami and Makino [4] (Figure 1); The large segment (L-segment) of RVFV has a single open reading frame (ORF) in the viral complementary sense coding region for L-protein (viral polymerase) [5]. The M-segment encodes two envelope glycoproteins, Gn and Gc, and two uncharacterized polypeptides (one expressed by himself (NSm1), and the other one in combination with Gn (NSm2)) [3,8]. The S-segment of RVFV has a unique ambisense coding strategy [24]. It contains two ORF; one encodes the nucleoprotein in the 3’ half of viral complementary sense molecule and the other encodes non-structural *NSs* protein in the 5’ half of viral sense RNA. The two ORFs are responsible for evading the host immune system, and they are separated by a short C-rich intergenic region. [7,25].

## 3. Epidemiology of RVF

### 3.1. RVF Disease Host Susceptibility

The most susceptible hosts to RVF infection are sheep, goats, lambs, cattle, buffaloes, some wild animals, and mice [1]. Camels may play an important role in the entrance and maintenance of RVFV in Egypt as camels enter Egypt via Sudan without any virological investigation or even a period of quarantine. Abortion storms in camels have been reported during RVF outbreaks in Egypt [26], and the virus was isolated from healthy and naturally infected camel blood. Interestingly, the importation of camel and sheep from Sudan was considered the main source of the RVF during the first Egyptian outbreak in 1977 [27]. Additionally, horses, pigs, fowl, and guinea pigs are resistant to RVFV infection. Especially during livestock outbreaks, RVFV caused serious disease in humans [28]. The age of the animal plays an important role in an animal’s susceptibility to a severe form of RVF [29]. For example, the mortality rate in lamb and adult sheep was 90% and 20–60%, respectively, while the abortion in pregnant ewes can reach 100% [30]. The disease was less severe in cattle and camels with a 10–30% mortality rate and the abortion rate in pregnant cows was 30–40% [31].

### 3.2. Risk Factors

RVF outbreaks in Egypt are associated with: (1) the importation of a large number of animals, especially the Sudanese dromedary camels via Sudan during the religious feasts (Sacrifice Feast), (2) environmental influences such as rainfall, and river discharge, which are considered important risk factors for RVF outbreaks in both animals and humans, (3) the availability of mosquito habitats due to the presence of the River Nile, (4) the high prevalence of different mosquito species that transmit the RVFV in Egypt, (5) recent RVF activity and vaccine usage, and (6) the consumption or handling of products from sick animals. The risk of human infection with RVF has increased in several occupational groups such as veterinarians, farmers, butchers, and animal handlers [32].

### 3.3. RVFV Vectors and Transmission Cycle

RVF virus has been isolated from several vectors including mosquitoes, ticks, and other flies [33,34]. There are about 19 culicine mosquito species that are distributed in different Egyptian provinces and are considered the main vector and source for RVFV transmission [32]. Mosquito genera such as *Aedes*, *Culex*, *Anopheles*, and *Mansonia* can transmit the virus [35]. The vectors of RVF virus are classified into maintenance vectors (*Aedes*) and amplifying vectors (*Culex*) [1]. *Aedes* species present in temporary flooded ground pools can acquire the infection through feeding on infected animal’s blood. The virus can survive on eggs of *Aedes* mosquitoes during inter-epizootic periods [36]. The eggs then require a period of dehydration, and they hatch into young virus-infected mosquitoes after heavy rainfall [1]. The *Culex* mosquitoes are involved in the transmission cycle once the virus is transmitted and replicated in host animals [36]. Both *Cx pipiens* and *Cx antennatus* are considered as natural vectors of RVF virus.

Notably, each of RVF epidemics in Egypt was caused by a different mosquito species. For example, during the epidemic of 1977, *Cx. pipiens* and *Cx. antennatus* were involved in the transmission of RVFV in El-Sharquiya province [37]; while during the 1993 epidemic, *Ae. caspius* acted as the primary RVFV vector; however, *Cx. pipiens*, *Cx. antennatus*, and *Cx. perexiguus* were also involved in this epidemic [38]. *Ae. caspius*, *Cx. perexiguus*, *Cx. pipiens* and *Cx. antennatus* were estimated to transmit 20%, 11%, 7%, and 7% of the RVF infection, respectively [39,40]. Most of the mosquitoes that transmitted RVFV during the 1993 epidemic were zoophagic rather than anthropophagic that fed on large animals (bovines, ovines, and equines) rather than humans [37,40]. Additionally, during the 2003 epidemic, six species of mosquitoes were involved in the virus transmission in Kafr El-Sheikh province (*Cx. antennatus* was the predominant species; 95.8%). However, the entomologic investigations characterized three RVFV isolates out of 297 tested female mosquito’s pools, and all three cases came from *Cx. antennatus*. It was considered the first report for natural infection of *Cx. antennatus* [41].

**Figure 2:**
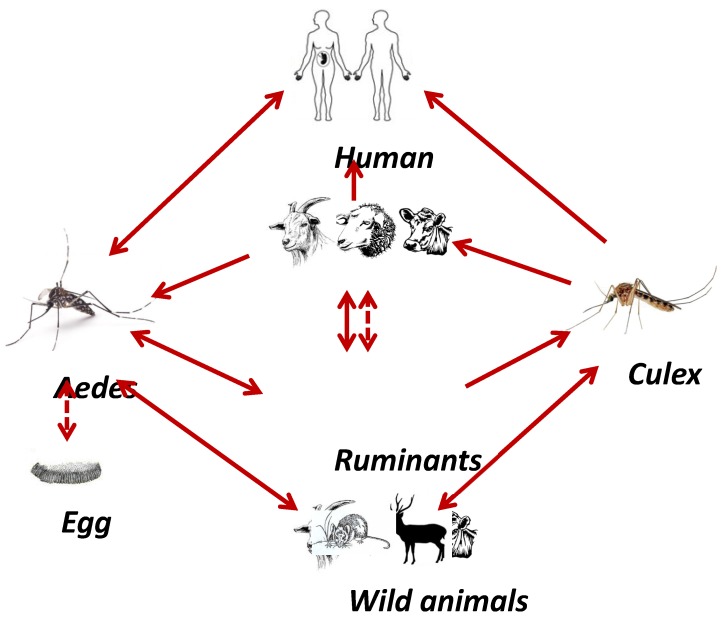
The life cycle of the RVF virus in Egypt and the role of the mosquito vectors. There are two transmission cycles for RVFV in nature: (1) an enzootic cycle that can occur during the normal rainfall and involves the *Aedes* mosquitoes, which transmit the virus vertically to their offsprings, and (2) an epidemic-epizootic cycle that occurs during abnormally heavy rainfall and flooding of dams or during the warm season. The virus is transmitted transovarially and the *Culex* mosquitoes distribute the virus and induce the emergence of outbreaks. The transmission of the virus to humans occurs by direct contact with infected animals. The continuous line represents the direct transmission, while the dashed line represents the vertical transmission.

There are two existing cycles of RVFV perpetuation in nature: (1) an enzootic cycle that occurs in the enzootic area of Africa during normal rainfall and (2) epidemic-epizootic cycle. During the first cycle, RVFV is present in a silent infection cycle emerges after rainfall to start the disease epizootics again. During this cycle, the *Aedes* mosquitoes transmit the virus vertically to their offspring. During the epidemic-epizootic cycle, the mosquitoes transmitted the virus transovarially. This cycle occurs during abnormal heavy rainfall and flooding of dams. The *Culex* mosquitoes distribute the virus and induce the emergence of outbreaks. [36]. The transmission cycle of RVF among animals, humans, and vectors is shown in Figure 2.

### 3.4. Mode of RVF Virus Transmission to Humans

Humans can acquire RVFV infection via a bite from an infected mosquito, contact with infected blood, tissue or body fluids. Infection can also occur during the killing, skinning, and cutting of infected animals, contact with the contaminated placenta, fetal and maternal blood of aborted animals, and consumption of raw milk or uncooked meat from infected animals [15].

### 3.5. Rift Valley Fever in Animals

Abortion storm is the main characteristic feature of RVF in pregnant animals. Infected animals might abort at any stage of gestation period due to the direct effect of the virus on the fetus; the abortion rate can reach 100% [1]. Morbidity and mortality rates vary according to the age and the species of animals. The mortality rate ranged from 70% to 100% in young animals [42]. In young animals the disease is associated with fever, anorexia, and ultimately death; whereas in adult animals, it varies from in-apparent form to acute form and is characterized by fever, weakness, bloody diarrhea, and vomiting [43].

### 3.6. Rift Valley Fever in Humans

RVFV infections in humans are asymptomatic or self-limiting [1,44]. The RVF disease starts after 4–6 days of an incubation period accompanied by some symptoms that include fever, chills, weakness, headache, as well as joint and muscle pain. These symptoms are followed by jaundice, painful eyes, diarrhea, vomiting, and insomnia [14,45,46]. RVFV infection is also associated with bleeding, low hemoglobin concentrations, lower platelet counts, rash, and malaise [17]. Approximately 1–2% of the cases suffer from severe complications that include: (1) neurological complications such as headache, irritation, confusion, coma encephalitis, and visual hallucination [46], (2) ocular complications including retinitis and vision loss [47], and (3) hemorrhagic fever with liver abnormalities which are characterized by fever, myalgia, and severe hemorrhage from mucous membrane [48]. There is a significant association between miscarriage in pregnant women with fevers and acute RVFV infection [17].

## 4. Endemicity of RVF Virus in Egypt

RVF is an endemic disease with a great economic impact in Egypt. There are several major factors that control the circulation and persistence of RVFV in Egypt, which are directly associated with One Health approach:

### 4.1. Vector-Associated Factors

The presence of an appropriate environment for the multiplication and growth of the vector mosquito, the wide distribution of mosquitoes in the Nile Valley and Delta with the absence of effective vector control programs that can interrupt the transmission cycle of the virus between arthropods and vertebrate hosts [2], and vertical transmission of the virus from female mosquitoes to their offspring. RVFV is transmitted by several species (more than 30 species) of mosquitoes that already present in Egypt [49].

### 4.2. Host-Associated Factors

Keeping the domestic animals in close contact with households, handling freshly slaughtered infected sheep meat, and a lack of public health education, has resulted in massive losses of human lives during RVF outbreaks. Additionally, other factors can increase the risk of human RVF infection, such as age (old more than young), sex, occupation (through contact with animal blood or body fluids), water, nutrition, socioeconomic status and poor sanitation [50,51]. The presence of host animals in Egypt such as camels, wild animals, and unvaccinated susceptible livestock [37], the continuous importation of animals, especially camels, from Africa, Sudan, and enzootic countries [37], and the slaughtering of sick animals for human consumption, which can easily spread RVFV infection via infected meat [52] contribute to RVF infection in humans.

### 4.3. Environmental and Climate Factors

The environment and climate directly impact how, when and where humans, animals, and vectors live and thrive. The presence of an appropriate environment for multiplication and growth of vector mosquito species, such as the presence of water ponds in the Egyptian villages, which can serve as a suitable environment for the breeding of mosquitoes, and the blood feeding habit of the vectors on sheep blood, an important reservoir host of RVFV, impact RVF transmission. Past outbreaks of RVFV have been associated with periods of heavy rainfalls. Fluctuations between drought and heavy rains in Egypt, leading to water collection near homes that serve as a suitable environment for mosquitoes breeding and make it easier to feed without flying so far from breeding sites, have contributed to RVFV outbreaks. The geographical location of Egypt in Africa and in the center of the Middle East maintains the endemicity of the disease; Egypt serves as a focal point that transmits the RVFV disease to Europe and Asia.

### 4.4. Additional Factors

The vaccination of RVF using RVF live attenuated vaccine (Smithburn’s strains) and an inactivated vaccine, which gave partial protection, alteration between vaccination programs (between inactivated and live vaccine) [53], and uncontrolled field trials of RVFV live attenuated strain, resulted in contamination of the environment, further influence RVFV transmission [52,54].

## 5. Epidemics of RVF in Egypt (Major Outbreaks)

**Figure 3:**
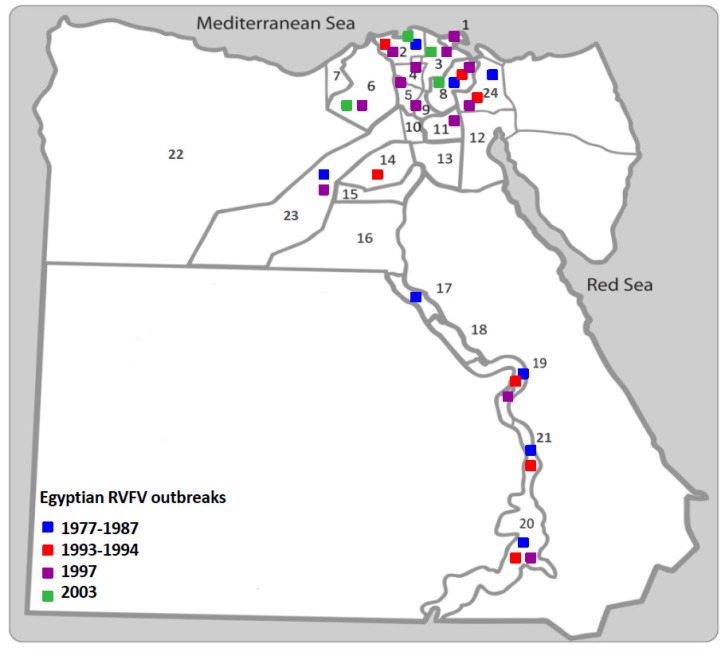
Distribution of RVF epidemics in Egypt. Egypt map shows different Egyptian provinces including: (1) Damietta, (2) Kafr El-Sheikh, (3) El-Dakahlia, (4) El-Gharbia, (5) El-Menofia, (6) El-Beheira, (7) Alexandria, (8) El-Sharquia, (9) El-Qalubia, (10) six of October, (11) Cairo (12) El-Suez, (13) Helwan, (14) El-Faiyoum, (15) Beni-Suef, (16) El-Menia, (17) Assiut, (18) Sohaj, (19) Qena, (20) Aswan, (21) Luxur, (22) Marsa Matrouh, (23) Giza, and (24) Ismailia. Blue dots represent the first outbreak (1977–1978), red dots represent the 1993–1994 outbreak, purple dots represent the 1997 outbreak, and the green dots represent the 2003 outbreak.

Five major RVF epidemics were reported in Egypt; 1977–1978, 1993, 1994, 1997, and 2003 [15] (Figure 3, Table 1):

### 5.1. The 1st Outbreak (1977)

The largest epizootic outbreak in Egypt resulted in severe losses (200,000 human infections and 600 deaths). During this epidemic, the virus was not only isolated from humans, but also from domestic animals, and rats (*Rattus rattus frugivorous*). During this outbreak, the first reported case was in Belbies, a city in El-Sharquia province in a man who suffered from an acute febrile dengue-like illness [52]. Previous studies suggested that the Egyptian troops who returned back after a peacekeeping mission in Congo might have transmitted the disease to the Belbies city because the outbreak appeared later in the same place [56]. Other studies suggested that RVFV was introduced to Egypt via the importation of infected animals, especially camels from Sudan, through the Aswan province [27]. However, a recent study confirmed that the Egyptian circulating strain was introduced from Zimbabwe as they are antigenically close related to RVFV strains that isolated from Zimbabwe in 1974 [55,59]. Most of the RVFV isolates were obtained from sheep, whereas only single isolate was obtained from each of other tested species such as cows, camels, horses, goats, and rats [52]. Massive human losses during Egyptian first RVF outbreak were due to: (1) a lack of the public background about the RVFV infection in Egypt, including clinical signs, vector, risk factors, and mode of transmission, (2) insufficient health education programs in Egypt about the RVFV and the required actions during outbreaks, (3) the absence of One Health program to control the disease, and (4) the high susceptibility of children and immunocompromised patients to RVFV infection. The disease reappeared again in the Aswan province of Egypt in 1983, and the reported seroprevalence in buffaloes, sheep, cattle, camels, goats, and equines were 35.3%, 22%, 14%, 7.3%, 6%, and 5.3%, respectively [57].

### 5.2. The 2nd Outbreak (1993)

This outbreak was recorded in the Aswan province with abortion storms in cattle, buffaloes and visual impairment in humans [60]. During this outbreak, the infection spread from the South to the North of Egypt in most of the Nile Delta and El-Faiyum provinces [27]. RVF was reported by the Animal Health Research Institute (AHRI) in animal farms located in Damietta province that used the live attenuated RVFV vaccine 20 days before the occurrence of the outbreak. Interestingly, the isolated viruses from the 1977 and the 1993 epidemics were phylogenetically similar [59]. This means that the virus was either reintroduced in 1993 from the same source (Sudan) or remained endemic between the two outbreaks. The RVF symptoms were detected by the Egyptian General Organization for Veterinary Services (GOVs) in imported pregnant cows and calves from South Africa after vaccination with the RVF live attenuated Smithburn vaccine.

### 5.3. The 3rd Outbreak (1994)

During this outbreak, RVFV has been isolated from 139 (31.7%) cattle and 84 (57.1%) sheep from Kafr El-Sheikh and El-Beheira provinces. The vaccination program using the locally produced or imported live attenuated Smithburn RVF vaccine failed to protect animals against RVF during this outbreak due to inadequate vaccination coverage [62].

### 5.4. The 4th Outbreak (1997)

This outbreak has occurred three years later to the previous epizootics in 1994, indicating the absence of an effective control and the failure of the vaccination program in Egypt using the live attenuated Smithburn strain vaccine during the previous outbreak. This outbreak began in Upper Egypt with abortion rates of 60–70% and 30–40% in pregnant ewes and cows respectively. The reported mortality rate was 50–60% in young lambs, 25–35% in adult sheep, 25–30% in calves and 10–20% in adult cattle [52]. A concurrent infection of theileriosis was reported with RVF infection on a dairy cattle farm in the Assiut province [52].

### 5.5. The 5th Outbreak (2003)

During this outbreak, RVF infection appeared in four Egyptian provinces located in the Nile Delta, including; Kafr El-Sheikh, El-Dakahliya, El-Beheira, and El-Sharquiya. The seroprevalence of RVF in 101 veterinary samples collected from Kafr El-Sheikh province was 10.4% and 5% in cattle and sheep, respectively [41]. Interestingly, this province was considered the main market for livestock animals in Egypt, where animals were collected from all Egyptian provinces. This was considered as a predisposing factor for this outbreak [41]. Additionally, acute febrile illness was recorded in Egyptian hospitals in 9 provinces with 29 confirmed RVF cases (out of 375 tested human samples). The highest prevalence of RVF (7.7%) was observed in Kafr El-Sheikh province [41]. The Naval Medical Research Unit No.3 (NAMRU-3) in Egypt confirmed that all cases were isolated from Egyptian farmers. The NAMRU-3 provided assistance during this outbreak based on a request from the Egyptian Ministry of Health and the WHO. However, the WHO reported an increase in RVF suspected cases in Kafr El-Sheikh province by active surveillance [63]. Further, the AHRI isolated RVFV from Damietta province during the same year. Three isolates were recovered from 297 tested pools of female mosquitoes, and all of them were isolated from *Cx. Antennatus* [41].

### 5.6. Since 2008

There has been no routine surveillance system for RVFV since 2008 and only sporadic investigations by veterinarians were recorded. Recently, a surveillance study has been conducted in several provinces in Egypt using different diagnostic methods [64]. The researchers found that the highest antibody level against RVF was detected in vaccinated cattle, buffaloes, sheep, and goats using serum neutralization test (SNT), agar gel precipitation test (AGPT), and enzyme linked immune sorbent assay (ELISA) from El-Menofia and Marsa Matrouh province with prevalence ranged between 68–80% and 80–93%, respectively. However, the lowest levels of antibodies (8.9–17.8%) were reported in vaccinated sheep from El-Qalubia province. The researchers also reported the prevalence of RVFV among non-vaccinated cattle, buffaloes, sheep, goats, and camels. They found that the coverage rates against RVFV were 2.8–4.8%, 10–15%, 14–19%, and 24–30% in El-Qalubia, Kafr El-Sheikh, El-Dakahlia, and El-Sharquia provinces, respectively [64].

Furthermore, antibodies against RVFV during the inter-epidemic period (2014 to 2015) were detected using different diagnostic methods such as ELISA, virus neutralization test (VNT), and indirect immunofluorescence assay (IIFA). The investigated animals (sheep, goats, buffaloes, and camels) were non-vaccinated, born after the last recorded Egyptian RVF epidemic in 2003, and were not imported from RVF endemic countries. The seroprevalence was 0%, 0.5%, 3.2%, and 5.9% in goats, sheep, camels, and buffaloes with total seroprevalence of 2.3% in all tested animals. This indicated the presence of a new RVF epidemic [65].

## 6. RVFV Surveillance in Egypt

Surveillance is the collection of information to help decision-makers take the appropriate actions to control RVFV. RVF surveillance activities include forecasting, early warning, and risk indicators, raising awareness, a reporting system, outbreak investigations, environmental surveillance, vector surveillance, and sentinel herds. The presence of several RVF outbreaks in Egypt was attributed to the lack of regular surveillance. Additionally, control of RVFV in Egypt is also limited due to difficult vector control, the absence of licensed human vaccine in Egypt, and availability of either inactivated vaccines with limited efficacy or live attenuated vaccines with reversion to virulence, and abortion storms in pregnant animals [66]. The best ways to prevent the entrance of RVF to Egypt or maintenance of RVF infection are; the developing and enhancing the regional monitoring system, establishing early warning and detection system, and using the One Health approach, which might help in the disease prediction and detection in animals before it is transmitted to humans. Egyptian veterinary authorities tried to apply these measures to prevent the introduction and spread of RVFV; however, some deficiencies still exist [52]. They are required to assess risk, build risk-targeted surveillance, and response capacity. Major RVFV outbreaks in animals precede human epidemics so the surveillance system of the disease should be started by One Health decision makers six months before the occurrence of the first human case [67]. There are some indicators of an RVF outbreak such as: (1) climatic change, (2) heavy prolonged rainfalls, (3) presence of widespread flooding, (4) increase mosquitos’ populations, (5) outbreaks of human febrile disease, and (6) hospital confirmed human case [68].

There are three surveillance methods for RVF based on OIE definition: (1) syndromic surveillance which is based on the detection of clinical cases or disease patterns. It depends on the presence of abortion storms in ruminants with early deaths (in lambs, kids or calves) especially during the presence of surface flooding following heavy rainfalls, (2) participatory surveillance which detects the best methods that help people to identify, solve their health needs and understand the risk perception with a greater involvement of animal owners in RVF disease surveillance. Participatory epidemiology is used in disease surveillance, risk assessment, disease control, disease recovery, prevention of reinfection, project development and epidemiological research. Participatory epidemiologists have developed qualitative risk maps as a first step for the RVF disease control, and (3) risk-based surveillance, which is surveillance of locations, populations, and periods with increased disease threats. It is aimed to detect disease rapidly and result in better use of resources. Risk-based surveillance uses quantitative or qualitative information (developed within several hours) that help in disease control [68].

Notably, many actions have been taken by the Egyptian Ministry of Health (MOH) before, during and after RVFV outbreaks which were considered as part of One Health approach such as: (1) testing of imported animals, (2) quarantine and vaccination of imported animals, (3) vaccination of susceptible animals before and during outbreaks occurring in humans, (4) RVFV disease notification, (5) daily reporting of RVF and encephalitis cases in humans, (6) surveillance and regular visits to fever and ophthalmic hospitals, (7) training of health care specialists on the early detection and management of RVFV cases, (8) providing the required diagnostic and research institutes with RVFV diagnostic tools, and (9) collaboration with the GOVs to evaluate the RVFV preventive programs [26,32].

## 7. Vaccination and Vaccine Development

Outbreaks of RVF indicated that human RVF infections were caused by contact with infected livestock, which is considered as an intermediate host between the vector and the humans [69]. Therefore, routine vaccination is considered as the cornerstone in controlling RVFV infections in animals in endemic countries to prevent human infections, socioeconomic loss, and disease outbreaks. The humoral immunity is sufficient for the protection against RVF infections [70], and the importance of cell-mediated immunity is still unknown. For example, the newborn lambs can acquire protection against RVF through the colostrums of immunized ewes [71]. There are no licensed vaccines for humans in Egypt, while there are three licensed veterinary vaccines used to protect ruminants against RVFV infections, including one live attenuated virus vaccine, and two inactivated virus vaccine. The ideal RVF vaccines must be efficient, safe, stable, elicit a rapid humoral immune response, induce long-term protection, and have low cost. Most of the RVFV vaccines are experimental and have only been tested in laboratory animals.

### 7.1. Inactivated RVFV Vaccines

The first RVF vaccine was formalin-inactivated and was developed from RVFV Entebbe strain (NDBR103), isolated from Uganda mosquitoes via 176 intraperitoneal or intravenous serial passages in mice, then 184 passages in green monkey kidney cells [72]. Interestingly, the NDBR103 vaccine was used for human vaccination in 1977 [73]. It was also used for 963 UN soldiers who received the vaccine administered subcutaneously (s/c) in three doses at 0, 7–10, and 28–30 days. The immunized soldiers exhibited good antibody titers, which reached its peak at six weeks post-vaccination [74]. The formalin-inactivated vaccine prepared from primary rhesus or African green monkey kidney cells is more immunogenic than vaccines prepared from chicken embryo cell culture [72]. TSI-GSD200 is a new generation of formalin-inactivated RVFV vaccine manufactured by USAMRIID via two passages of Entebbe strain in diploid cells (FRHL-2 cells) of the fetal rhesus monkey lung cells [73]. This vaccine was used for vaccinating service troops by three s/c injections on days 0, 7, and 28, followed by a single booster dose after six-months. The vaccine produces a 1:237 neutralizing antibody titer and induced long-term immunity in vaccinated humans with primer and booster doses [75]. Recently, formalin or binary ethylenamine inactivated RVFV vaccine was prepared only for veterinary use via passage on baby hamster kidney (BHK-21) cells [73]. The NDBR 103 and TSI-GSD 200 vaccines caused swelling, erythema, tenderness, or pain at the site of inoculation without systemic febrile reaction [71]. Interestingly, the Veterinary Serum and Vaccine Research Institute (VSVRI) in Egypt developed another formalin-inactivated vaccine from Egyptian RVFV strains that were isolated from the 1977 outbreak (ZH501). The virus was propagated in BHK-21 cells, inactivated with 0.5% formalin with aluminum hydroxide adjuvant, and used only for animal immunization in Egypt [76]. Repeated immunization and high cost are the main disadvantages of inactivated vaccines [73].

### 7.2. Live-Attenuated RVFV Vaccines

#### 7.2.1. Smithburn Vaccine

The Smithburn live attenuated RVFV vaccine is one of the oldest and widely used vaccines for the control of RVF infections in Egypt. It was isolated in 1944 in Uganda from a mosquito *Eretmapodites* spp. The neurotropic RVFV strain was generated in South Africa between 1953 and 1985 via 102 serial passages in mouse brain, 54 passages in embryonated chicken egg and another 16 passages in mouse brain. In 1958, only 103 serial passages were performed for the RVFV in mouse brain and elicited better protection [59]. Since 1971, this vaccine has been propagated via passages in BHK-21 cells to produce modified live virus vaccine (MLVV) for the vaccination of livestock in susceptible African countries, such as South Africa, Kenya, Saudi Arabia and Egypt [71]. The same virus was used to produce live-attenuated vaccines in Kenya and Egypt in 1960 and 1994 respectively. In spite of its potency and low cost, the Smithburn vaccine has several disadvantages including residual pathogenicity, abortions, fetal malformations, and reversion to virulence. Therefore, the live attenuated vaccine is prohibited to be used in pregnant animals and restricted to be used in the RVFV free countries [76]. There is also a possibility of reassortment when the vaccine used during outbreaks, leading to increased diversity of the virus [9]. This vaccine has been reported to cause pathological changes in liver of kids and abortion in pregnant ewes. Therefore the researcher has concluded that it is not safe to be used in Egypt which considered an endemic area [54]. The Smithburn vaccine provides protection after a single administration and induces long-lasting protective immunity against RVFV.

#### 7.2.2. MP-12 Live Attenuated Vaccine

The MP-12 vaccine was developed by the U.S. Army Medical Research Institute of Infectious Diseases (USAMRIID) for both human and veterinary use via 12 serial passages of the virulent ZH548 and ZH501 strains, isolated from Egyptian patients in MRC-5 cells in the presence of chemical mutagens (5-fluorouracil). MP-12 is temperature sensitive and carries redundant mutations in all three genomic segments of RVFV [77]. Furthermore, reassortants between the wild-type strain and MP-12 showed that these segments contribute to the attenuation of MP-12 virulence in mice [78]. The reassortant strains contain one attenuated and two virulent segments that considered to be attenuated, therefore, the reassortment between wild-type virus and vaccine strain might produce an attenuated virus that protects against RVF [78]. The MP-12 vaccine was evaluated in pregnant ewes at 70–100 days of gestation. Interestingly, MP-12 did not induce abortions, fetal abnormalities or virus shedding in milk when administered after 3 months of gestation. Only some lamb losses (about 4%) were aborted when the MP-12 vaccine was administered at the early stage of pregnancy [79]. This induces neutralizing antibodies from 1:80 to 1:320 titers, and the newborn lambs acquire more than 1:80 neutralizing antibodies after they are fed colostrums via the passive transfer of maternal antibodies. The MP-12 vaccine was also evaluated for human use in Rhesus Macaques and produced protective antibody titers (≥1:40) against challenge test with RVFV ZH501 strain [80]. Generally, MP-12 has the ability to produce antibody titers sufficient to protect the animals against RVFV infection and there is a potential beneficial effect of immunizing pregnant animals to obtain protection in newborns. The MP-12 vaccine contains virulent *NSs* gene and can cause abortions and fetal abnormalities, but the vaccine is well tolerated, safe, and immunogenic when administered at an adequate dose. The MP-12 vaccine gave promising results when evaluated in more than 100 human volunteers [71,81].

#### 7.2.3. Naturally Attenuated Clone 13 Vaccine

The natural deletion of 549 nucleotides (70%) of the *NSs* gene (the main virulence factor), leads to the development of attenuated clone 13 vaccine. This vaccine is a plaque-derived clone, isolated from immunocompetent patients infected with 74HB59 RVFV strain from the Central African Republic [82,83]. Clone 13 vaccine is highly potent (equivalent to Smithburn vaccine), safe, causes no abortions or teratogeny in immunized pregnant ewes [84,85,86], and did not cause detectable viremia in vaccinated ruminants, which reduces the risk of virus transmission to the fetus or to mosquito vectors [84]. However, neurological signs in experimentally vaccinated mice were reported [87,88], suggesting that the vaccine is not completely harmless. Further, administration of the pregnant ewes with an overdose of Clone 13 vaccine in the first trimester, allowing the vaccine to cross the placental barrier, leading to fetal malformations, and stillbirth [89].

Clone 13 vaccinated animals can be easily differentiated from naturally infected animals using DIVA test, as the immunized animals do not elicit antibodies against the deleted antigen (NSs). The mutant virus is unable to replicate efficiently in vaccinated animals and does not produce a long-term immune response, which is the main disadvantages of the vaccine. The Clone 13 vaccine has been licensed in some countries, including South Africa, Kenya, Botswana and Namibia [73]. Furthermore, CL13T, a thermostable clonal isolate of Clone 13 has been developed. This vaccine was safe and immunogenic in sheep, cattle, goats and pregnant camels [90,91].

The reassortment of clone-13 and MP-12 vaccines via passage together on Vero cells resulted in the presence of live attenuated R566 vaccine. This vaccine contains S-segment of the clone-13 virus with an attenuated mutation in M- and L-segment of the MP-12 virus [49]. This vaccine induced partial protection and still needs numerous improvements.

### 7.3. Recombinant Virus Vaccines

#### 7.3.1. Virus- Vectored vaccine

There are many virus vectors that have been identified as vaccines for controlling RVFV. For example, heterologous vectors expressing Gn and/or Gc glycoproteins and non-structural proteins of RVFV and they include: (1) Vaccinia virus Copenhagen strain (Vco) devoid of the virulent gene, which expresses Gn and Gc of RVFV, elicit protective antibody titers, and is highly safe in mice, primates, and ruminant [92], (2) Lumpy skin disease virus (LSDV) which was experimentally evaluated in South Africa as a vector for RVFV glycoproteins and can generate neutralizing antibodies against LSDV, RVFV, and sheep poxvirus (which is closely related capripoxvirus) [93,94], (3) Chimpanzee adenovirus construct vector (ChAdOx1-GnGc) which also carry Gn and Gc of RVFV can induce humoral and cell-mediated immunity and provided complete protection against RVFV infection in ruminants [95,96], (4) Modified Vaccinia virus Ankara (MVA) which express RVFV envelope glycoproteins Gn and Gc and showed efficacy in murine models [97], (5) Recombinant New Castle disease virus (NDV) that was evaluated in calves and induced neutralizing antibodies in sheep after two doses [98,99]. Sheep and cattle are not the natural hosts of Newcastle virus, leading to the absence of preexisting neutralizing antibodies in vaccinated animals, which is the main advantage of NDV vectored-vaccine, (6) alphavirus (Sindbis) replicon vaccine, which exhibited 100% protection against RVFV in mice, and augmented RVFV neutralizing antibody responses in sheep [100], and (7) equine herpesvirus type 1 (EHV-1) vector vaccine, which induced RVFV neutralizing antibody titers in sheep [101]. Recombinant RVFV vaccines lacking *NSs* and *NSm* proteins can overcome fetal malformations and abortions [102]. The Virus-vectored vaccine possessed high efficacy and safety in both pregnant and non-pregnant sheep [102].

#### 7.3.2. Plasmid DNA Vaccine

Plasmid DNA vaccines have been used against some pathogens when scientists discovered that naked DNA could be transferred into tissue and express the antigen [103]. DNA vaccines encoding a protective antigen with a strong promoter can be used to express protective proteins [104]. Administration of plasmid DNA vaccine is usually via intramuscular, intradermal, or mucosal routes and reaches the host cells via endocytic vesicles, membrane pores, or mediated receptors [105]. Inside the cells, DNA is transferred to the nucleus and is presented to the immune system [106]. A DNA vaccine was developed from plasmid encoding, Gn and Gc of RVFV, Hantavirus, Crimean-Congo hemorrhagic fever virus, and *preM* and E genes of tick-borne encephalitis virus [107]. The DNA vaccine combination produced protective antibodies against RVFV infection in mice; whereas DNA vaccine expressing Gn and Gc exhibited a 20% survival rate in mice [94]. This vaccine is stable, suitable for use in tropical countries and has variable efficacy based on gene gun immunization of mice with a plasmid carrying G1 and G2 of RVFV [107,108]. The low immunogenicity of DNA vaccines can be enhanced by using immune modulators [66] or using booster dose of the DNA vaccine or primary vaccination with a plasmid DNA vaccine followed by a booster dose with attenuated or viral vectored vaccine [109].

#### 7.3.3. Virus-Like Particles Based Vaccine

Virus-like particles (VLPs) is a replication-deficient viral particle that consists of the viral envelope protein (Gn and Gc), and N protein while lacking the viral infectious genetic material [110,111]. The VLPs of RVFV were made from viral nucleoprotein, glycoproteins, or both [110,111,112] The VLPs are characterized by (1) a lack of the risk of virus replication and inactivation, (2) a morphological similarity to the virulent virus, (3) enhanced adsorption with cell wall and digesting in endolysosomes, (4) the stimulation of both MHC class I and class II responses, (5) the stimulation of cellular and humoral-mediated immune responses against RVFV [113], and (6) antigen stability and immunogenicity as it produces neutralization antibody titer of 1:250 to 1:1250 in immunized mice and passes a challenge test against RVFV ZH548 strain [110,114,115]. The main disadvantage of the VLP vaccine is costs associated with mass production.

#### 7.3.4. Glycoprotein-Based Subunit Vaccine

Subunit vaccines have the advantages of using viral protein or parts of viral protein which enhance the protective immune response. The subunit vaccine contains Gn/Gc and possesses a neutralizing antibody response to protect sheep and lambs against RVFV infection [116]. Additionally, Gn protein expressed in eukaryotic insect cells has enhanced immunity in lambs [98]. The nucleoprotein N of RVFV can be used for the development of subunit vaccine; however, it provides a partial immune response with a 60% protection of mice using a challenge test [94]. The vaccination with pure and crude RVFV surface glycoprotein induced strong neutralizing antibodies that completely protect against a challenge test. The subunit vaccine is highly safe for use in endemic areas and DIVA vaccine, but is poorly immunogenic and requires the use of booster doses [117,118].

#### 7.3.5. Reverse Genetic Vaccine

The reverse genetic technology system helps in the management of complementary DNA (cDNA) copies of each RVFV genome segment in vitro for the generation of novel reverse genetic vaccines [49,119,120,121,122]. The production of attenuated recombinant RVFV vaccine has been established by a reverse genetic system (infectious clone system) [3,110,123,124]. Laboratory studies carried out on the immune-evading role of non-structural viral proteins allow the creation of virus deficient for these proteins using reverse genetics technology. The virulent vaccines were developed and examined in rats and ruminants [102,125,126,127]. Reverse genetic systems were used to generate new ZH501-derived mutant virus (rRVF-ΔNSs:GFP-ΔNSm ) with M-segment lacking the *NSm* gene and containing green fluorescence protein (GFP) in the *NSs* gene of the S-segment [102]. The rRVF-ΔNSs:GFP-ΔNSm virus is highly attenuated (due to the lack of *NSs*) and is highly immunogenic in rats challenged with RVFV at 28 days post vaccination after a single 1 × 10^3^ or 1 × 10^4^ pfu vaccination [102]. Additionally, DDvax vaccine (recombinant ZH501 strain), that encoding the deletions of *NSs* and *NSm* genes [rZH501-ΔNSs-ΔNSm] has shown protective efficacy in pregnant ewes, without causing any adverse effects in newborn lambs [125]. The reverse genetic vaccines have shown protective and immunogenic effects in sheep, rats, and mice without producing clinical signs. The single deletion mutant (ΔNSs rRVFV) and double deletion mutant (ΔNSs-ΔNSm rRVFV) vaccines were shown to be immunogenic and safe in the marmoset (Callithrix jacchus), a non-human primate (NHP) model. These vaccines induced strong antibody response after a single dose without adverse reactions, clinical signs, or detection of infectious virus in vaccinated marmosets. Also, there were no detectable viremia or liver diseases in animals challenged with RVFV [128]. RVFV vaccines lack *NSs* gene and/or *NSm* gene with the insertion of a nonviral gene is useful to differentiate between naturally infected and vaccinated animals (DIVA test) as the vaccinated animals elicit antibodies against the inserted nonviral gene and do not elicit antibodies against deleted proteins [71].

## 8. Prevention and Control of RVF Disease in Egypt

Once RVFV is established in a free area, it is difficult to be eradicated because of: (1) the presence of several routes of transmission, (2) the presence of several mosquito species that can transmit the virus, and (3) the replication of the virus inside the mosquito.

### 8.1. For Humans

There is no safe and licensed commercial vaccine available for the control of RVFV infection in humans until now. Some trials were conducted to produce safe human vaccines such as inactivated vaccines, including NDBR-103, [72] and TSI-GSD 200 [71].

### 8.2. For Animals

The control of RVFV in animals in Egypt was based on detection of the disease, vector control, restriction of animal movement, and animal vaccination. Several vaccines were reported to be used for the control of RVF in Egypt including: (1) Formalin-inactivated vaccine with alum adjuvant (Menya/sheep/258) strain produced by VACSERA company in Egypt, (2) Binary ethylenamine inactivated vaccine with alum adjuvant (ZH501 RVF) strain, produced by Veterinary Serum and Vaccines Research Institute (VSVRI), and (3) Live Smithburn neurotropic attenuated strain produced by VSVRI [52]. The live-attenuated RVFV vaccine (Smithburn vaccine) is a Freeze-dried vaccine and produced using Vero cells at a concentration of 10^4^ TCID 50/mL [52]. This vaccine has several disadvantages including (1) it is used only for immunization of sheep and goats (2) it is unsafe for pregnant animals and can cause abortions and birth defects, (3) it cannot be used during the breeding season of mosquitoes, which can extend to 12 months in Egypt due to ecological and environmental factors such as a warm winter, (4) vaccinated animals cannot be slaughtered within 21 days of vaccination, (5) not all vaccinated animals give a good antibody response to this vaccine, and (6) potential for reversion of RVFV to virulence in vaccinated animals [54,76].

In addition, the formalin or binary ethylenamine inactivated whole virion vaccine conjugated with aluminum hydroxide adjuvant also has some disadvantages including: (1) the vaccine preparation and the vaccination process is laborious, (2) the vaccine requires a long production time, (3) it requires biosafety level 3 laboratories, and (4) it needs annual booster doses which make it expensive and not practical in routine animal field vaccinations [82]. Interestingly, researchers compared Egyptian locally produced live attenuated and inactivated RVFV vaccines by using field trials on lambs and calves in Alexandria province of Egypt. They found that the inactivated vaccine was safe and gave similar protection in comparison to the live attenuated vaccine, especially with a booster dose administered after five months [129].

Zagazig H501 inactivated strain was used to vaccinate RVFV susceptible animals such as cattle, buffaloes, sheep, goats, and camels. About 5.9 million doses of RVFV vaccines were used in 2014 compared to 7.3 million doses used in 2015 [26]. The RVFV vaccine coverage was 60%, 35%, 30%, and 30% for cattle, buffaloes, sheep, and goats respectively. Furthermore, the vaccine coverage in Egypt varies according to the province. For example, the vaccine coverage in cattle was 20% in Assiut province, 30% in the El-Sharquia, El-Behira, and Beni-Suef provinces [26].

The Egyptian GOVs have banned the use of live attenuated-RVFV (Smithburn vaccine) since 2007 and have decided to use only the inactivated ZH501 vaccine for regular immunization against RVFV in animals. The inactivated vaccine has overcome the disadvantages of the live attenuated vaccine, such as abortion in pregnant animals and reversion to virulence. The GOVs have obtained the vaccine from the VSVRI, and immunize the animals for free.

## 9. Vaccination Regime of the Imported Animals to Egypt

Egypt has been frequently affected by RVF outbreaks which are mainly associated with the importation of animals from endemic sub-Saharan countries. Therefore, vaccination of the animals before importation or upon their arrival would help in the control of RVF epidemics. Between 2012 and 2015, approximately 762,291 camels were legally imported to Egypt: 79.4% from Sudan and 20.6% from Ethiopia. However, approximately 149,943 cattle were legally imported to Egypt: 67% from Sudan and 33% from Ethiopia [26]. Interestingly, cattle were slaughtered in quarantine facilities (no imported cattle were allowed to be released alive from the quarantine), while the camels were released from the quarantine to slaughterhouses or to animal markets. There was a low risk of RVFV transmission by vectors in many quarantine facilities including (1) Abu Simbel quarantine, where camels and cattle were imported from Sudan, (2) Sahl Hasheesh quarantine, where cattle were imported from Ethiopia, (3) Shelateen quarantine, where camels were imported from Eastern Sudan, and (4) Adabiya quarantine, where camels imported from Ethiopia. However, there was a high risk of RVFV transmission by vectors in Al Qata quarantine, where camels from Ethiopia arrived [26].

Imported camels from Sudan usually enter the Abu Simbel and Shalateen facilities, remain in quarantine for 3 days and are vaccinated against RVFV upon arrival; while the imported camels from Ethiopia remained in Adabiya or Al-Qata quarantines for 10–16 days and received the first dose of RVFV vaccine 7 days before shipment to Egypt and a booster dose was administered upon arrival to the Egyptian quarantines. Additionally, imported cattle from Sudan received the first dose of RVFV vaccine in Sudan, whereas imported cattle from Ethiopia received the first dose of vaccine at Djibouti and the booster dose upon arrival to the Egyptian quarantine [26].

## 10. Conclusion, Future Prospective and One Health Approach

RVFV is a highly fatal endemic viral disease in Egypt and was included by the OIE in the category A- diseases that have a potential of rapid and extensive spread. This disease causes severe economic losses in humans and animals in Egypt. RVF is characterized by complex epidemiology due to the involvement of multiple mosquito species in the transmission cycle. Therefore, this disease is a significant problem that requires collaboration between multidiscipline researchers from different organizations to control the disease in Egypt. In order to effectively control the disease in Egypt, we recommend an effective collaboration between GOVs, the ministry of health, epidemiologists, veterinarians, and stakeholders. Currently, the One Health approach is used for the management and understanding of animal, human, and environmental determinants of the disease through the collaboration and direct communication between veterinarians, occupational health physicians, and public health operators in order to control the RVF through improving the education system, status of thinking, legislation, and administrative structures. Recently, the One Health approach can save up to 35% of RVFV control costs [21].

One Health approach of RVFV requires collaboration among: (1) medical and veterinary clinicians, diagnosticians, epidemiologists, and public health experts to perform rapid diagnosis, notification, treatment of the affected patients and animals, provide notices to minimize the risk of future disease spread among the affected species, (2) wildlife experts to understand the epidemiology of the disease in wildlife and its role in disease transmission, (3) entomologists to understand the vector biology, its role in RVFV epidemiology and to provide advice on vector control, (4) ecologists to determine the impact of the disease on the ecology and the environment, (5) economists and social scientists to assess the social and economic impact of RVF disease on the populations, (6) governments and decision makers to write policies and support funding required for the One Health approach, prevention, and control, (7) vaccinologists to develop and provide the vaccines for virus control, and (8) pharmaceutical industries for treatment (both human and animals) and vector control (repellents, insecticides, acaricides, and larvicides). The One Health approach includes: (1) using a safe and effective RVFV vaccine to vaccinate the susceptible animals under the supervision of the government in order to decrease outbreaks in humans, (2) the establishment of a strong and regular surveillance system and fast reporting program for the disease, (3) the hiring of well trained and professional personnel, (4) conducting epidemiological investigations to determine the risk factors, (5) the training of veterinary and medical health specialists on the diagnosis and management of suspected cases, (6) increasing public health awareness about the RVFV and its transmission, (7) breaking the virus transmission cycle by controlling the mosquitoes, and (8) controlling animal importation and quarantine for imported animals from Africa.

The World Health Organization (WHO) recommendations during RVF outbreaks are: (1) avoiding contact with infected animals especially their body fluids directly or via aerosols, which is considered as the most significant risk factor for RVF virus, (2) practicing hand washing, and wearing gloves to control animal-to-human transmission of RVFV, (3) avoiding consumption of infected raw milk or animal tissue, (4) using protective measures to avoid mosquito bites by using mosquito nets, personal insect repellent, light, and wearing colored clothing (long-sleeved shirts and trousers), and (5) avoiding outdoor activity such as camping at the peak biting times of the vectors mosquito in order to reduce human infection and deaths [29]. In conclusion, the One Health approach is required to mitigate outbreaks of RVF in Egypt. Further, the collaboration between veterinary, health, and environmental authorities both on national and regional levels is needed to control RVFV infection in Egypt.

## Figures and Tables

**Table 1:** Egyptian RVFV outbreaks.

Outbreaks	Presumable Source of Infection	Affected Areas	Main Vectors	Human Cases	Affected Animals	Used Vaccine	References
1977 (August–December) to 1978 (July–December)	The infected person returns back from Africa and importation of infected camels from Sudan-Zimbabwe	Belbies, El-Sharquia province, then spread to the Nile valley, delta, and Sudan	*Cx. Pipiens*	1977: up to 20,000 cases, with 598 deaths 1978: 114 confirmed cases, with 12 deaths	Domestic animals (sheep, cattle, camels, goats, horses), rats and humans	No available vaccines	[27,37,52,53,55,56,57,58,59]
1993–1994 (May–August)	The virus either remains endemic after 1977 outbreak or reintroduced in 1993 from the same source (Sudan)	Aswan, then spread to Nile Delta provinces, El-Faiyum and Damietta	*Ae. Caspius*	Up to 1500 estimated cases, with 128 confirmed cases	Domestic animals (cattle and buffaloes) and humans	Live attenuated Smithburn strain	[27,38,52,60,61]
1997 (April–August)	Importation of animals especially camels from Africa with the absence of effective control measure	Upper Egypt, then spread to all Egyptian provinces	-	7 confirmed cases	Domestic animals (sheep and cattle) and humans	Live attenuated Smithburn strain	[52]
2003 (June–October)	RVFV appeared in the main market of livestock animals in Egypt, where animals were collected from all over the country	Began to appear in four provinces (Kafr El-Sheikh, El-Sharquiya, El-Dakahliya and El-Beheira) in the Nile Delta	*Cx. Antennatus*	373 confirmed cases, with 112 deaths	Domestic animals (cattle and sheep) and humans	Live attenuated Smithburn strain	[41,52]

## Abbreviations

AHRI	Animal Health Research Institute
Ae	*Aedes*
ARBO	Arthropod-borne
BEI	Binary ethylenimine
BHK-2	Baby hamster kidney
ChAdOx1-GnGc	Chimpanzee adenovirus construct vector
CL13T	Thermostable clonal isolate of Clone 13
Cx	*Culex*
DIVA	Differentiation between infected and vaccinated animals
EHV-1	Equine herpesvirus type 1
ELISA	Enzyme-Linked Immune-Sorbent Assay
FRHL-2 cells	Fetal rhesus monkey lung cells
GFP	Green fluorescence protein
GOVs	Egyptian General Organization for Veterinary Services
IIFA	Indirect immunofluorescence assay
kDa	Kilo Dalton
LSDV	Lumpy skin disease virus
L segment	Large segment
M segment	Medium segment
MOH	Egyptian Ministry of Health
MLVV	Modified live virus vaccine
MVA	Modified Vaccinia virus Ankara
NAMRU-3	Naval Medical Research Unit No.3
NDV	New Castle Disease Virus
NHP	Non-human primate
NSm	Non-structural M protein
NSs	Non-structural protein
OIE	Office international des epizootes
ORF	Open reading frame
RVF	Rift Valley fever
RVFV	Rift Valley fever virus
cDNA	Complementary DNA
rRVF-ΔNSs:GFP-ΔNSm	ZH501-Derived mutant RVF virus
S/C	Subcutaneously
spp.	Species
S segment	Small segment
TCID50	Tissue Culture Infective Dose50
UN	United Nations
USAMRIID	U.S. Army Medical Research Institute of Infectious Diseases
Vco	Vaccinia virus Copenhagen strain
VLPs	Virus-like particles
VNT	Virus Neutralization Test
VSVRI	Veterinary Serum and Vaccines Research Institute
WHO	World Health Organization
$	US Dollars

## References

[1] Pepin M., Bouloy M., Bird B.H., Kemp A., Paweska J. (2010). Rift valley fever virus (*Bunyaviridae: Phlebovirus*): An update on pathogenesis, molecular epidemiology, vectors, diagnostics and prevention. Vet. Res..

[2] Moutailler S., Roche B., Thiberge J.-M., Caro V., Rougeon F., Failloux A.-B. (2011). Host alternation is necessary to maintain the genome stability of rift valley fever virus. PLoS Negl. Trop. Dis..

[3] Gerrard S.R., Nichol S.T. (2007). Synthesis, proteolytic processing and complex formation of N-terminally nested precursor proteins of the rift valley fever virus glycoproteins. Virology.

[4] Ikegami T., Makino S. (2011). The pathogenesis of rift valley fever. Viruses.

[5] Muller R., Poch O., Delarue M., Bishop D.H., Bouloy M. (1994). Rift valley fever virus L segment: Correction of the sequence and possible functional role of newly identified regions conserved in RNA-dependent polymerases. J. Gen. Virol..

[6] Billecocq A., Spiegel M., Vialat P., Kohl A., Weber F., Bouloy M., Haller O. (2004). Nss protein of rift valley fever virus blocks interferon production by inhibiting host gene transcription. J. Virol..

[7] Gauliard N., Billecocq A., Flick R., Bouloy M. (2006). Rift valley fever virus noncoding regions of L, M and S segments regulate RNA synthesis. Virology.

[8] Suzich J., Kakach L., Collett M. (1990). Expression strategy of a phlebovirus: biogenesis of proteins from the rift valley fever virus M segment. J. Virol..

[9] Ikegami T. (2012). Molecular biology and genetic diversity of rift valley fever virus. Antivi. Res..

[10] Daubney R., Hudson J., Garnham P. (1931). Enzootic hepatitis or rift valley fever. An undescribed virus disease of sheep cattle and man from east Africa. J. Pathol. Bacteriol..

[11] Bird B.H., Ksiazek T.G., Nichol S.T., Maclachlan N.J. (2009). Rift valley fever virus. J. Am. Vet. Med. Assoc..

[12] Rich K.M., Wanyoike F. (2010). An assessment of the regional and national socio-economic impacts of the 2007 rift valley fever outbreak in Kenya. Am. J. Trop. Med. Hyg..

[13] Al-Afaleq A., Hussein M. (2011). The status of rift valley fever in animals in Saudi Arabia: A mini review. Vector Borne Zoonotic Dis..

[14] Madani T.A., Al-Mazrou Y.Y., Al-Jeffri M.H., Mishkhas A.A., Al-Rabeah A.M., Turkistani A.M., Al-Sayed M.O., Abodahish A.A., Khan A.S., Ksiazek T.G. (2003). Rift valley fever epidemic in Saudi Arabia: Epidemiological, clinical, and laboratory characteristics. Clin. Infect. Dis..

[15] Helmy Y.A., El-Adawy H., Abdelwhab E.M. (2017). A comprehensive review of common bacterial, parasitic and viral zoonoses at the human-animal interface in Egypt. Pathogens.

[16] Borio L., Inglesby T., Peters C.J., Schmaljohn A.L., Hughes J.M., Jahrling P.B., Ksiazek T., Johnson K.M., Meyerhoff A., O’Toole T. (2002). Hemorrhagic fever viruses as biological weapons: Medical and public health management. JAMA.

[17] Baudin M., Jumaa A.M., Jomma H.J.E., Karsany M.S., Bucht G., Naslund J., Ahlm C., Evander M., Mohamed N. (2016). Association of rift valley fever virus infection with miscarriage in Sudanese women: A cross-sectional study. Lancet Glob. Health.

[18] Hassan O.A., Affognon H., Rocklov J., Mburu P., Sang R., Ahlm C., Evander M. (2017). The one health approach to identify knowledge, attitudes and practices that affect community involvement in the control of rift valley fever outbreaks. PLoS Negl. Trop. Dis..

[19] Drake J.M., Hassan A.N., Beier J.C. (2013). A statistical model of rift valley fever activity in Egypt. J. Soc. Vector Ecol..

[20] McElroy A., Albariño C.G., Nichol S.T. (2009). Development of a RVFV Elisa that can distinguish infected from vaccinated animals. Virol. J..

[21] Rostal M.K., Ross N., Machalaba C., Cordel C., Paweska J.T., Karesh W.B. (2018). Benefits of a one health approach: An example using rift valley fever. One Health.

[22] Maes P., Alkhovsky S.V., Bao Y., Beer M., Birkhead M., Briese T., Buchmeier M.J., Calisher C.H., Charrel R.N., Choi I.R. (2018). Taxonomy of the family Arenaviridae and the order Bunyavirales: Update 2018. Arch. Virol..

[23] Ikegami T., Makino S. (2004). Rift valley fever virus. Viruses.

[24] Giorgi C., Accardi L., Nicoletti L., Gro M.C., Takehara K., Hilditch C., Morikawa S., Bishop D.H.L. (1991). Sequences and coding strategies of the S RNAs of Toscana and rift valley fever viruses compared to those of Punta Toro, Sicilian Sandfly fever, and Uukuniemi viruses. Virology.

[25] Brennan B., Welch S., Elliott R. (2014). The consequences of reconfiguring the Ambisense S genome segment of rift valley fever virus on viral replication in mammalian and mosquito cells and for genome packaging. PLoS Pathog..

[26] Napp S., Chevalier V., Busquets N., Calistri P., Casal J., Attia M., Elbassal R., Hosni H., Farrag H., Hassan N. (2018). Understanding the legal trade of cattle and camels and the derived risk of rift valley fever introduction into and transmission within Egypt. PLoS Negl. Trop. Dis..

[27] Corwin A., Habib M., Watts D., Olson J., Darwish M., Hibbs R., Kilpatrick M. (1993). Prevalence of antibody to rift valley fever virus in the Nile river delta of Egypt, 13 years after a major outbreak. Trans. R. Soc. Trop. Med. Hyg..

[28] Weiss K. (1957). Rift valley fever a review. Bull. Epizoot. Dis. Afr..

[29] WHO (2018). Rift Valley Fever. http://www.Who.Int/news-room/fact-sheets/detail/rift-valley-fever.

[30] Budasha N.H., Gonzalez J.P., Sebhatu T.T., Arnold E. (2018). Rift valley fever seroprevalence and abortion frequency among livestock of kisoro district, south western Uganda (2016): A prerequisite for zoonotic infection. BMC Vet. Res..

[31] Allam I.H., Feinsod F.M., Scott R.M., Peters C.J., Saah A.J., Ghaffar S.A., El Said S., Darwish M.A. (1986). Rift valley fever surveillance in mobile sheep flocks in the Nile delta. Am. J. Trop. Med. Hyg..

[32] Kenawy M.A., Abdel-Hamid Y.M., Beier J.C. (2018). Rift valley fever in Egypt and other African countries: Historical review, recent outbreaks and possibility of disease occurrence in Egypt. Acta. Trop..

[33] Davies F.G., Koros J., Mbugua H. (1985). Rift valley fever in Kenya: The presence of antibody to the virus in camels (camelus dromedarius). J. Hyg. (Lond.).

[34] Labuda M., Nuttall P. (2004). Tick-borne viruses. Parasitology.

[35] Diallo D., Ba Y., Dia I., Lassana K., Diallo M. (2008). Use of insecticide-treated cattle to control rift valley fever and west Nile virus vectors in Senegal. Bull. Soc. Pathol. Exot..

[36] Lumley S., Horton D.L., Hernandez-Triana L.L.M., Johnson N., Fooks A.R., Hewson R. (2017). Rift valley fever virus: Strategies for maintenance, survival and vertical transmission in mosquitoes. J. Gen. Virol..

[37] Gad A.M., Riad I.B., Farid H.A. (1995). Host-feeding patterns of *culex pipiens* and *cx. antennatus* (diptera: Culicidae) from a village in Sharqiya governorate, Egypt. J. Med. Entomol..

[38] Turell M.J., Presley S.M., Gad A.M., Cope S.E., Dohm D.J., Morrill J.C., Arthur R.R. (1996). Vector competence of Egyptian mosquitoes for rift valley fever virus. Am. J. Trop. Med. Hyg..

[39] Kenawy M.A., Beier J.C., Zimmerman J.H., el Said S., Abbassy M.M. (1987). Host-feeding patterns of the mosquito community (diptera: Culicidae) in Aswan governorate, Egypt. J. Med. Entomol..

[40] Gad A.M., Farid H.A., Ramzy R.R., Riad M.B., Presley S.M., Cope S.E., Hassan M.M., Hassan A.N. (1999). Host feeding of mosquitoes (diptera: Culicidae) associated with the recurrence of rift valley fever in Egypt. J. Med. Entomol..

[41] Hanafi H.A., Fryauff D.J., Saad M.D., Soliman A.K., Mohareb E.W., Medhat I., Earhart K.C. (2011). Virus isolations and high population density implicate *culex* antennatus (becker) (diptera: Culicidae) as a vector of rift valley fever virus during an outbreak in the Nile delta of Egypt. Acta. Tropica..

[42] Gerdes G. (2010). Rift valley fever, the veterinary clinics of North America. Food Anim. Pract..

[43] Busquets N., Xavier F., Martin-Folgar R., Lorenzo G., Galindo-Cardiel I., del Val B.P., Rivas R., Iglesias J., Rodriguez F., Solanes D. (2010). Experimental infection of young adult European breed sheep with rift valley fever virus field isolates. Vector Borne Zoonotic Dis..

[44] Archer B.N., Thomas J., Weyer J., Cengimbo A., Landoh D.E., Jacobs C., Ntuli S., Modise M., Mathonsi M., Mashishi M.S. (2013). Epidemiologic investigations into outbreaks of rift valley fever in humans, South Africa, 2008–2011. Emerg. Infect. Dis.

[45] LaBeaud A., Muchiri E., Ndzovu M., Mwanje M., Muiruri S., Peters C., King C.H. (2008). Interepidemic rift valley fever virus seropositivity, northeastern Kenya. Emer. Infect. Dis..

[46] Seufi A., Galal F. (2010). Role of *culex* and *anopheles* mosquito species as potential vectors of rift valley fever virus in Sudan outbreak, 2007. BMC Infect. Dis..

[47] Yoser S.L., Forster D.J., Rao N.A. (1993). Systemic viral infections and their retinal and choroidal manifestations. Surv. Ophthalmol..

[48] Muiruri S., Peters C.J., Clinton White A., LeDuc J., King C.H., Njenga M.K., Muchiri E.M. (2010). Severe rift valley fever may present with a characteristic clinical syndrome. Amer. J. Trop. Med. Hyg..

[49] Kortekaas J., Oreshkova N., van Keulen L., Kant J., Bosch B.J., Bouloy M., Moulin V., Goovaerts D., Moormann R.J. (2014). Comparative efficacy of two next-generation rift valley fever vaccines. Vaccine.

[50] El-Mekki A.A. (2014). Female gender: A risk factor for acquiring rift valley fever virus infection in Jazan region, southwestern Saudi Arabia. Med. J. Cairo Uni..

[51] Nyakarahuka L., de St Maurice A., Purpura L., Ervin E., Balinandi S., Tumusiime A., Kyondo J., Mulei S., Tusiime P., Lutwama J. (2018). Prevalence and risk factors of rift valley fever in humans and animals from kabale district in southwestern Uganda, 2016. PLoS Negl. Trop. Dis..

[52] Kamal S.A. (2011). Observations on rift valley fever virus and vaccines in Egypt. Virol. J..

[53] Meegan J.M. (1979). The rift valley fever epizootic in Egypt 1977–1978. Description of the epizzotic and virological studies. Trans. R Soc. Trop Med. Hyg..

[54] Kamal S.A. (2009). Pathological studies on postvaccinal reactions of rift valley fever in goats. Virol. J..

[55] Samy A.M., Peterson A.T., Hall M. (2017). Phylogeography of rift valley fever virus in Africa and the Arabian peninsula. PLoS Negl. Trop. Dis..

[56] Mahmoud A., Ibrahim MK F.A. (1989). Rift valley fever: Pathological studies on suspected heifers from Friesian dairy farm with a history of abortion. Egypt J. Comp. Pathol. Clin. Pathol..

[57] Imam I., El Karamany R., Omar F., El Kafrawi O. (1981). Rift valley fever in Egypt. J. Egypt. Public Health Assoc..

[58] El-Akkad A.M. (1978). Rift valley fever outbreak in Egypt. October–December 1977. J. Egypt Public Health Assoc..

[59] Grobbelaar A.A., Weyer J., Leman P.A., Kemp A., Paweska J.T., Swanepoel R. (2011). Molecular epidemiology of rift valley fever virus. Emerg. Infect. Dis..

[60] Arthur R.R., el-Sharkawy M.S., Cope S.E., Botros B.A., Oun S., Morrill J.C., Shope R.E., Hibbs R.G., Darwish M.A., Imam I.Z. (1993). Recurrence of rift valley fever in Egypt. Lancet.

[61] Taha M., Elian K., Marcoss T., MS Eman A.L. (2001). Monitoring of rift valley fever virus in Egypt during year 2000 using Elisa for detection to both IgM and IgG specific antibodies. J. Egypt Vet. Med. Ass..

[62] Ghoneim N.H., Woods G.T. (1983). Rift valley fever and its epidemiology in Egypt: A review. J. Med..

[63] WHO (2018). Disease Outbreak Reported: Rift Valley Fever in Egypt. https://www.who.int/news-room/fact-sheets/detail/rift-valley-fever.

[64] Marawan M.A., Ebied M.H., Galila E.M., Youssef A.I., Hassan K.Z. (2012). Epidemiological studies on rift valley fever disease in Egypt. Benha Vet. Med. J..

[65] Mroz C., Gwida M., El-Ashker M., El-Diasty M., El-Beskawy M., Ziegler U., Eiden M., Groschup M.H. (2017). Seroprevalence of rift valley fever virus in livestock during inter-epidemic period in Egypt, 2014/15. BMC Vet. Res..

[66] Boshra H., Lorenzo G., Busquets N., Brun A. (2011). Rift valley fever: Recent insights into pathogenesis and prevention. J. Virol..

[67] Jost C.C., Nzietchueng S., Kihu S., Bett B., Njogu G., Swai E.S., Mariner J.C. (2010). Epidemiological assessment of the rift valley fever outbreak in Kenya and Tanzania in 2006 and 2007. Am. J. Trop. Med. Hyg..

[68] Mariner J. Rift Valley Fever Surveillance. Fao Animal Production And Health Manual No. 21. http://www.Fao.Org/3/i8475en/i8475en.Pdf.

[69] Bird B.H., Nichol S.T. (2012). Breaking the chain: Rift valley fever virus control via livestock vaccination. Curr. Opin. Virol..

[70] Niklasson B.S., Meadors G.F., Peters C.J. (1984). Active and passive immunization against rift valley fever virus infection in Syrian hamsters. Acta. Pathol. Microbiol. Immunol. Scand. C.

[71] Ikegami T., Makino S. (2009). Rift valley fever vaccines. Vaccine.

[72] Randall R., Gibbs C.J., Aulisio C.G., Binn L.N., Harrison V.R. (1962). The development of a formalin-killed rift valley fever virus vaccine for use in man. J. Immunol..

[73] Faburay B., LaBeaud A.D., McVey D.S., Wilson W.C., Richt J.A. (2017). Current status of rift valley fever vaccine development. Vaccines (Basel).

[74] Niklasson B., Peters C.J., Bengtsson E., Norrby E. (1985). Rift valley fever virus vaccine trial: Study of neutralizing antibody response in humans. Vaccine.

[75] Pittman P.R., Liu C.T., Cannon T.L., Makuch R.S., Mangiafico J.A., Gibbs P.H., Peters C.J. (1999). Immunogenicity of an inactivated rift valley fever vaccine in humans: A 12-year experience. Vaccine.

[76] Botros B., Omar A., Elian K., Mohamed G., Soliman A., Salib A., Salman D., Saad M., Earhart K. (2006). Adverse response of non-indigenous cattle of European breeds to live attenuated Smithburn rift valley fever vaccine. J. Med. Virol..

[77] Caplen H., Peters C.J., Bishop D.H. (1985). Mutagen-directed attenuation of rift valley fever virus as a method for vaccine development. J. Gen. Virol..

[78] Saluzzo J.F., Smith J.F. (1990). Use of reassortant viruses to map attenuating and temperature-sensitive mutations of the rift valley fever virus mp-12 vaccine. Vaccine.

[79] Morrill J.C., Mebus C.A., Peters C.J. (1997). Safety and efficacy of a mutagen-attenuated rift valley fever virus vaccine in cattle. Am. J. Vet. Res..

[80] Morrill J.C., Peters C.J. (2011). Protection of mp-12-vaccinated rhesus macaques against parenteral and aerosol challenge with virulent rift valley fever virus. J. Infect. Dis..

[81] Mansfield K.L., Banyard A.C., McElhinney L., Johnson N., Horton D.L., Hernandez-Triana L.M., Fooks A.R. (2015). Rift valley fever virus: A review of diagnosis and vaccination, and implications for emergence in europe. Vaccine.

[82] Kortekaas J., Oreshkova N., Cobos-Jimenez V., Vloet R.P., Potgieter C.A., Moormann R.J. (2011). Creation of a nonspreading rift valley fever virus. J. Virol..

[83] Muller R., Saluzzo J.F., Lopez N., Dreier T., Turell M., Smith J., Bouloy M. (1995). Characterization of clone 13, a naturally attenuated avirulent isolate of rift valley fever virus, which is altered in the small segment. Am. J. Trop. Med. Hyg..

[84] Dungu B., Louw I., Lubisi A., Hunter P., von Teichman B.F., Bouloy M. (2010). Evaluation of the efficacy and safety of the rift valley fever clone 13 vaccine in sheep. Vaccine.

[85] von Teichman B., Engelbrecht A., Zulu G., Dungu B., Pardini A., Bouloy M. (2011). Safety and efficacy of rift valley fever smithburn and clone 13 vaccines in calves. Vaccine.

[86] Njenga M.K., Njagi L., Thumbi S.M., Kahariri S., Githinji J., Omondi E., Baden A., Murithi M., Paweska J., Ithondeka P.M. (2015). Randomized controlled field trial to assess the immunogenicity and safety of rift valley fever clone 13 vaccine in livestock. PLoS Negl. Trop. Dis..

[87] Dodd K.A., McElroy A.K., Jones T.L., Zaki S.R., Nichol S.T., Spiropoulou C.F. (2014). Rift valley fever virus encephalitis is associated with an ineffective systemic immune response and activated t cell infiltration into the CNS in an immunocompetent mouse model. PLoS Negl. Trop. Dis..

[88] Vialat P., Billecocq A., Kohl A., Bouloy M. (2000). The s segment of rift valley fever phlebovirus (bunyaviridae) carries determinants for attenuation and virulence in mice. J. Virol..

[89] Makoschey B., van Kilsdonk E., Hubers W.R., Vrijenhoek M.P., Smit M., Wichgers Schreur P.J., Kortekaas J., Moulin V. (2016). Rift valley fever vaccine virus clone 13 is able to cross the ovine placental barrier associated with foetal infections, malformations, and stillbirths. PLoS Negl. Trop. Dis..

[90] Daouam S., Fakri F., Ennaji M., El arkam A., Tadlaoui K., Oura C., Elharrak M. (2014). Heat stability of the rift valley fever virus clone 13 live vaccines. Trials Vaccinol..

[91] Daouam S., Ghzal F., Arkam A.E., Naouli Y., Jazouli M., Ennaji M.M., Tadlaoui K.O., Oura C., El Harrak M. (2015). Evaluation of the safety and efficacy of a live attenuated thermostable rift valley fever vaccine in sheep, goats and cattle. J. Vaccines Vaccin..

[92] Papin J.F., Verardi P.H., Jones L.A., Monge-Navarro F., Brault A.C., Holbrook M.R., Worthy M.N., Freiberg A.N., Yilma T.D. (2011). Recombinant rift valley fever vaccines induce protective levels of antibody in baboons and resistance to lethal challenge in mice. Proc. Natl. Acad. Sci. USA.

[93] Soi R.K., Rurangirwa F.R., McGuire T.C., Rwambo P.M., DeMartini J.C., Crawford T.B. (2010). Protection of sheep against rift valley fever virus and sheep poxvirus with a recombinant capripoxvirus vaccine. Clin. Vaccine Immunol..

[94] Wallace D.B., Ellis C.E., Espach A., Smith S.J., Greyling R.R., Viljoen G.J. (2006). Protective immune responses induced by different recombinant vaccine regimes to rift valley fever. Vaccine.

[95] Warimwe G.M., Gesharisha J., Carr B.V., Otieno S., Otingah K., Wright D., Charleston B., Okoth E., Elena L.G., Lorenzo G. (2016). Chimpanzee adenovirus vaccine provides multispecies protection against rift valley fever. Sci. Rep..

[96] Warimwe G.M., Lorenzo G., Lopez-Gil E., Reyes-Sandoval A., Cottingham M.G., Spencer A.J., Collins K.A., Dicks M.D., Milicic A., Lall A. (2013). Immunogenicity and efficacy of a chimpanzee adenovirus-vectored rift valley fever vaccine in mice. Virol. J..

[97] Lopez-Gil E., Lorenzo G., Hevia E., Borrego B., Eiden M., Groschup M., Gilbert S.C., Brun A. (2013). A single immunization with MVA expressing GNGC glycoproteins promotes epitope-specific CD8+-T cell activation and protects immune-competent mice against a lethal RVFV infection. PLoS Negl. Trop. Dis..

[98] Kortekaas J., Antonis A.F., Kant J., Vloet R.P., Vogel A., Oreshkova N., de Boer S.M., Bosch B.J., Moormann R.J. (2012). Efficacy of three candidate rift valley fever vaccines in sheep. Vaccine.

[99] Kortekaas J., Dekker A., de Boer S.M., Weerdmeester K., Vloet R.P., de Wit A.A., Peeters B.P., Moormann R.J. (2010). Intramuscular inoculation of calves with an experimental newcastle disease virus-based vector vaccine elicits neutralizing antibodies against rift valley fever virus. Vaccine.

[100] Heise M.T., Whitmore A., Thompson J., Parsons M., Grobbelaar A.A., Kemp A., Paweska J.T., Madric K., White L.J., Swanepoel R. (2009). An alphavirus replicon-derived candidate vaccine against rift valley fever virus. Epidemiol. Infect..

[101] Said A., Elmanzalawy M., Ma G., Damiani A.M., Osterrieder N. (2017). An equine herpesvirus type 1 (EHV-1) vector expressing rift valley fever virus (RVFV) GN and GC induces neutralizing antibodies in sheep. Virol. J..

[102] Bird B.H., Albarino C.G., Hartman A.L., Erickson B.R., Ksiazek T.G., Nichol S.T. (2008). Rift valley fever virus lacking the NSS and NSM genes is highly attenuated, confers protective immunity from virulent virus challenge, and allows for differential identification of infected and vaccinated animals. J. Virol..

[103] Tang D.C., DeVit M., Johnston S.A. (1992). Genetic immunization is a simple method for eliciting an immune response. Nature.

[104] Rajcani J., Mosko T., Rezuchova I. (2005). Current developments in viral DNA vaccines: Shall they solve the unsolved?. Rev. Med. Virol..

[105] Wolff J.A., Budker V. (2005). The mechanism of naked DNA uptake and expression. Adv. Genet..

[106] Belekova J., Horynova M., Krupka M., Weigl E., Raska M. (2007). DNA vaccines: Are they still just a powerful tool for the future?. Arch. Immunol. Ther. Exp..

[107] Spik K., Shurtleff A., McElroy A.K., Guttieri M.C., Hooper J.W., SchmalJohn C. (2006). Immunogenicity of combination DNA vaccines for rift valley fever virus, tick-borne encephalitis virus, hantaan virus, and crimean congo hemorrhagic fever virus. Vaccine.

[108] Lagerqvist N., Naslund J., Lundkvist A., Bouloy M., Ahlm C., Bucht G. (2009). Characterisation of immune responses and protective efficacy in mice after immunisation with rift valley fever virus cDNA constructs. Virol. J..

[109] Lorenzo G., Martin-Folgar R., Hevia E., Boshra H., Brun A. (2010). Protection against lethal rift valley fever virus (RVFV) infection in transgenic ifnar(-/-) mice induced by different DNA vaccination regimens. Vaccine.

[110] Habjan M., Penski N., Spiegel M., Weber F. (2008). T7 RNA polymerase-dependent and -independent systems for cdna-based rescue of rift valley fever virus. J. Gen. Virol..

[111] Liu L., Celma C.C., Roy P. (2008). Rift valley fever virus structural proteins: Expression, characterization and assembly of recombinant proteins. Virol. J..

[112] Pichlmair A., Habjan M., Unger H., Weber F. (2010). Virus-like particles expressing the nucleocapsid gene as an efficient vaccine against rift valley fever virus. Vector Borne Zoonotic Dis..

[113] Beyer T., Herrmann M., Reiser C., Bertling W., Hess J. (2001). Bacterial carriers and virus-like-particles as antigen delivery devices: Role of dendritic cells in antigen presentation. Curr. Drug Targ. Infect. Disord..

[114] Mandell R.B., Koukuntla R., Mogler L.J., Carzoli A.K., Holbrook M.R., Martin B.K., Vahanian N., Link C.J., Flick R. (2010). Novel suspension cell-based vaccine production systems for rift valley fever virus-like particles. J. Virol. Methods.

[115] Naslund J., Lagerqvist N., Habjan M., Lundkvist A., Evander M., Ahlm C., Weber F., Bucht G. (2009). Vaccination with virus-like particles protects mice from lethal infection of rift valley fever virus. Virology.

[116] Faburay B., Wilson W.C., Gaudreault N.N., Davis A.S., Shivanna V., Bawa B., Sunwoo S.Y., Ma W., Drolet B.S., Morozov I. (2016). A recombinant rift valley fever virus glycoprotein subunit vaccine confers full protection against rift valley fever challenge in sheep. Sci. Rep..

[117] Faburay B., Lebedev M., McVey D.S., Wilson W., Morozov I., Young A., Richt J.A. (2014). A glycoprotein subunit vaccine elicits a strong rift valley fever virus neutralizing antibody response in sheep. Vector Borne Zoonotic Dis..

[118] Hansson M., Nygren P.A., Stahl S. (2000). Design and production of recombinant subunit vaccines. Biotechnol. Appl. Biochem..

[119] Stobart C.C., Moore M.L. (2014). RNA virus reverse genetics and vaccine design. Viruses.

[120] Wichgers Schreur P.J., Oreshkova N., Moormann R.J., Kortekaas J. (2014). Creation of rift valley fever viruses with four-segmented genomes reveals flexibility in bunyavirus genome packaging. J. Virol..

[121] Chen H., Angel M., Li W., Finch C., Gonzalez A.S., Sutton T., Santos J., Perez D.R. (2014). All-in-one bacmids: An efficient reverse genetics strategy for influenza a virus vaccines. J. Virol..

[122] Hu B., Jiang J., Zhan J., Li G., Jiang Y., Guan X., Chen Y., Fang Z. (2014). Development of a reverse genetics system for respiratory syncytial virus long strain and an immunogenicity study of the recombinant virus. Virol. J..

[123] Billecocq A., Gauliard N., Le May N., Elliott R.M., Flick R., Bouloy M. (2008). Rna polymerase I-mediated expression of viral RNA for the rescue of infectious virulent and avirulent rift valley fever viruses. Virology.

[124] Ikegami T., Won S., Peters C.J., Makino S. (2006). Rescue of infectious rift valley fever virus entirely from cDNA, analysis of virus lacking the NSS gene, and expression of a foreign gene. J. Virol..

[125] Bird B.H., Maartens L.H., Campbell S., Erasmus B.J., Erickson B.R., Dodd K.A., Spiropoulou C.F., Cannon D., Drew C.P., Knust B. (2011). Rift valley fever virus vaccine lacking the NSS and NSM genes is safe, nonteratogenic, and confers protection from viremia, pyrexia, and abortion following challenge in adult and pregnant sheep. J. Virol..

[126] Morrill J.C., Laughlin R.C., Lokugamage N., Wu J., Pugh R., Kanani P., Adams L.G., Makino S., Peters C.J. (2013). Immunogenicity of a recombinant rift valley fever mp-12-nsm deletion vaccine candidate in calves. Vaccine.

[127] Weingartl H.M., Nfon C.K., Zhang S., Marszal P., Wilson W.C., Morrill J.C., Bettinger G.E., Peters C.J. (2014). Efficacy of a recombinant rift valley fever virus mp-12 with nsm deletion as a vaccine candidate in sheep. Vaccine.

[128] Smith D.R., Johnston S.C., Piper A., Botto M., Donnelly G., Shamblin J., Albarino C.G., Hensley L.E., Schmaljohn C., Nichol S.T. (2018). Attenuation and efficacy of live-attenuated rift valley fever virus vaccine candidates in non-human primates. PLoS Negl. Trop. Dis..

[129] Youssef B. (2004). Study the immune response for sheep and cattle with rift valley fever inactivated and attenuated vaccine. Alex J. Med. Sci..

